# Editorial: Pharmacoepidemiology in Diabetes Mellitus and its Complications: Focus on Response to Medications

**DOI:** 10.3389/fphar.2022.917977

**Published:** 2022-05-24

**Authors:** Soroush Mohammadi Jouabadi, Tarunveer Singh Ahluwalia, Taulant Muka, Fariba Ahmadizar

**Affiliations:** ^1^ Division of Pharmacoepidemiology, Department of Epidemiology, Erasmus MC, University Medical Center, Rotterdam, Netherlands; ^2^ Division of Vascular Pharmacology, Department of Internal Medicine, Erasmus MC, University Medical Center, Rotterdam, Netherlands; ^3^ Steno Diabetes Center Copenhagen (SDCC), Gentofte, Denmark; ^4^ Department of Preclinical Medicine, Medical School, Institute for Social and Preventive Medicine, University of Bern, Bern, Switzerland; ^5^ Julius Global Health, University Medical Center Utrecht, Utrecht, Netherlands

**Keywords:** diabetes mellitus, pharmacokinetics, pharmacodynamics, markers, efficacy, glucose control

## Importance of Optimal Pharmacotherapy for Diabetes Mellitus

In light of pharmacotherapy with glucose-lowering medications (GLMs), not all diabetic patients maintain standard glucose levels, which may contribute to long-term complications, including but not limited to vascular damage. These complications can highly affect the quality of life of diabetic patients and are an extensive causal nexus for the increased burden of mortality and morbidity and treatment costs. Diabetic people with complications, on average, are charged 50–70% higher medical expenses juxtaposed to non-complicated diabetic cases (Chan et al., 2020; Williams et al., 2022). Therefore, understanding how these medications may play a role in preventing or increasing the risk of these complications is crucial in delivering precision medicine and treatment optimization. Improved therapeutic effectiveness under Pharmacokinetic/Pharmacodynamics (PK/PD) modeling and Pharmacoepidemiological studies will yield significant vigor and vitality and help patients keep their societal position.

## PK/PD and Pharmacoepidemiological Studies: Narrowing the Gap of Data Sources

PK/PD studies bear a shred of key evidence regarding intra-individual variations in the drug metabolism, distribution, and biotransformation and may relatively explain failures in response to medications. PK/PD studies usually follow stringent inclusion criteria for patient recruitment and hence are not generalizable to patients of clinical practice where intricate inter-variable interactions exist. On the other hand, longitudinal data of pharmacoepidemiological studies with longer follow-up spans provides better insight into assessing outcomes such as mortality, safety endpoints, and treatment trajectory, including dosage modifications, add-on or switching to another drug, and adverse outcomes drug reactions (ADR), treatment adherence and compliance. Moreover, increased accessibility to larger patient registries provided a great opportunity for PE studies to contribute efficiently to diabetes research by answering crucial clinical and policy inquiries (Salvo and Faillie, 2019). However, PK/PD studies would be preferable in drawing causal inferences. Accordingly, integrating the evidence from PK/PD and PE studies as two important branches of pharmacology could be the gold standard for better treatment optimization and decision-making.

## ERA for New Generation of Glucose-Lowering Medications?

While several studies have linked first-generation Sulfonylureas with an increased risk of cardiovascular events and death, there is a promising trend highlighting the potential of new GLMs generation, e.g., glucagon-like peptide 1 (GLP-1) analogs and sodium-glucose cotransporter 2 (SGLT2) inhibitors in preventing cardiovascular complications of diabetes patients (Scheen, 2018). The cardioprotective effects of these medications have been embedded through several mechanisms, preponderantly hemodynamic moderations. This evidence fabricates a novel standpoint for managing the cardiovascular complications of diabetic patients. Pharmacoepidemiological studies also help identify and validate potential inter and intra drug-drug interactions concerning new GLMs generation and their effect on patients’ health status. Generated evidence can inaugurate a pedestal for the future clinical decision-making of diabetes pharmacotherapy (Zhou et al., 2021). Notwithstanding, further efforts necessitate pointing out the superiority of these treatments to each other and whether these findings are generalizable to all diabetic patients. This has been reflected in the breadth of articles in this special issue, based on different data resources worldwide.

## In This Special Issue

This special issue delivers further vision through the effectiveness and safety of GLMs in diabetes control and its micro/macro-vascular obstacles by covering a wide range of race diversity, interventions, and existing co-morbidities. In this issue, Tye et al. predict the effect of SGLT2 inhibitors on the risk of cardiorenal outcomes. Furthermore, the efficiency of a pre-developed prediction score was evaluated compared to the observed outcomes, indicating that some unknown underlying mechanisms are yet to be captured to perfectly predict the effects of SGLT2 inhibitors Tye et al. In this regard, Seong et al. also denoted the superiority of GLP-1 analogs to Dipeptidyl Peptidase-4 inhibitors for their cardiorenal events protection, stratified the risk based on individuals with and without underlying co-morbidities (Seong et al.). Pooled results of existing evidence in either clinical trials or real-world settings also endorse the promising role of SGLT2 inhibitors in preventing cardiovascular events in diabetes patients. Over and above the safety and effectiveness of GLMs themselves (Liu et al.), the study by Moranges et al. scrutinized the effect modification role of several factors that may interact with the cardiovascular efficacy of SGLT2 inhibitors (Mortanges et al.). This special issue also advances evidence-based pharmacotherapy as an intervention and appraises the potential of health care providers in achieving diabetes control (Bukhsh et al.).

Furthermore, evidence demonstrates that several factors contribute to failure in initial diabetes pharmacotherapy and henceforth switching or addition to other GLMs. In this special issue, the study by Wood et al. applied an advanced pharmacoepidemiological approach to promoting our understanding of the diabetes management complexity where several determinants such as underlying co-morbidities, co-medications, and adherence to therapy may instigate switching or add-ons (Wood et al.).

Forging ahead, the field of diabetes pharmacoepidemiology is challenging with several limitations and gaps: lack of direct encounter effectiveness appraisal among different GLMs, methods to handle the multiplex real-world intra-variable interactions, methods to overcome several possible biases in this type of study, and unsatisfactory integration with PK/PD studies that can help elucidate the causal mechanisms linked to the failure of pharmacotherapy.

In toto, this special issue endeavors to amass the evidence encouraging the role of optimum pharmacotherapy in preventing diabetes complications, especially cardiorenal outcomes.
